# Hybrid genomes of three multidrug-resistant *Salmonella enterica* serovar Newport (REPJJP01) isolates from exotic captive felids in North Dakota, USA

**DOI:** 10.1128/mra.00021-26

**Published:** 2026-06-22

**Authors:** Daniel R. Evans, Kelli J. Maddock, Brianna L. S. Stenger

**Affiliations:** 1 North Dakota Health and Human Services 1749, Bismarck, North Dakota, USA; 2 Minnesota Department of Health 11055, St. Paul, Minnesota, USA; 3 Veterinary Diagnostic Laboratory, North Dakota State University 3323, Fargo, North Dakota, USA; University of Wisconsin-Madison, Madison, Wisconsin, USA

**Keywords:** *Salmonella*, multidrug resistance, genomes

## Abstract

Three *Salmonella enterica* isolates from captive exotic felines were sequenced on Illumina and Oxford Nanopore platforms. The hybrid assemblies showed the strains to be the persisting multidrug-resistant *Salmonella enterica* serovar Newport strain REPJJP01. Hosts were two snow leopards (*Panthera uncia*) and a Pallas’s cat (*Otocolobus manul*) from North Dakota zoological facilities.

## ANNOUNCEMENT

*Salmonella* is a significant and persistent pathogen of humans and animals. Raw meat diets are common for captive carnivores and are increasingly a common commercially available food choice for companion animals. Raw meat diets, including commercially prepared options, are also a significant risk factor for *Salmonella* exposure for humans (1, 2) and animals (3, 4) alike. Here we present complete, circularized genome assemblies of multidrug-resistant (MDR) *Salmonella enterica*, serovar Newport persisting strain REPJJP01 isolated from two snow leopards (*Panthera uncia*) and a Pallas’s cat (*Otocolobus manul*). These genomes importantly expand the host range and add to the literature for this persisting strain isolated from residents of all 50 states and previously associated with consumption of U.S. beef products and travel to or consumption of cheese from Mexico (5).

Feces or intestinal tissue for routine diagnostic testing were submitted to the North Dakota State University Veterinary Diagnostic Laboratory and cultured for enteric pathogens using routine procedures (6). Single colonies identified as *Salmonella* were serotyped as previously described (6, 7). To complement genotypic results, antimicrobial susceptibility testing was performed using Vitek 2 Compact GN-87 cards, gradient diffusion, and disk diffusion testing (6).

Total nucleic acids were extracted (MagMAX CORE kit; Applied Biosystems, Thermo Fisher Scientific, Waltham, MA, USA; cat. no. A32702) from isolated colonies inoculated in 200 μL of nuclease-free water (Growcells, San Diego, CA, USA; cat. no. NUPW-1000) following the manufacturer’s instructions and assessed with a DeNovix DS-11 spectrophotometer with a fluorometric FX module and dsDNA Broad Range Fluorescent Assay kit (DeNovix Inc., Wilmington, DE, USA; SKU K-30050). Short-read libraries were prepared using the Illumina DNA Prep Kit and 1/2 reagent volumes as described (8) and sequenced on an iSeq 100 (Illumina, San Diego, CA, USA). Long-read libraries were prepared using Oxford Nanopore Technologies’ (ONT, Oxford, United Kingdom) Rapid Barcoding Kit (SQK-RBK004), following the manufacturer's instructions, and sequenced on an ONT MinION (Mk1C) sequencer with a R9.4.1 flow cell (FLO-MIN106, ONT) and MinKNOW v22.12.5. Long-read base calling was performed by Guppy v6.4.6 in fast mode with reads below a *Q* score of 7 filtered out. Single and pooled libraries were assessed prior to sequencing using the DeNovix DS-11 and the dsDNA High Sensitivity Fluorescent Assay kit (DeNovix Inc., SKU K-30080). Quality control, species identification, short-read assembly, quality checks, and hybrid genome assembly were performed with FastQC v0.11.8 (9), Kraken2 v2.0.8 (10), Shovill v1.1.0 (11), QUAST v0.5.2 (12), and Unicycler v0.5.1 (13), respectively. MOB-typer v3.1.9 (14) identified plasmid contigs from the hybrid genomes, and AMRFinderPlus v4.0.19 (15) was used to detect AMR genes. Genomes were annotated by NCBI using the Prokaryotic Genome Annotation Pipeline (16). All tools used default settings. Genomic sequencing data and annotations are summarized in Table 1.

**TABLE 1:** Summary of sequencing results

Host source	1837Paun	21-7809Otma	22-2675Paun
	*Panthera uncia* (snow leopard)	*Otocolobus manul* (Pallas’s cat)	*Panthera uncia* (snow leopard)
Sample type	Intestine	Intestine	Feces
Collection year	2022	2021	2022
Collection location (U.S. state)	North Dakota	North Dakota	North Dakota
No. of total Illumina bases	174,682,139	219,523,571	132,073,906
No. of Illumina raw reads	613,478	771,164	462,618
Genome coverage, Illumina reads (×)	26	44	34
No. of ONT raw reads	169,237	326,472	137,275
No. of ONT total bases	1,230,711,779	2,107,296,260	1,014,779,251
No. of hybrid contigs (chromosome)	1	1	1
No. of hybrid contigs (plasmid)	2	1	1
Hybrid contig N50 size (bp)	4,845,634	4,880,147	4,879,466
Hybrid plasmid size (bp)	46,876 and 34,148	46,950	46,950
Plasmid incompatibility group	IncR	IncR	IncR
Hybrid total genome size (bp)	4,926,658	4,927,097	4,926,416
Hybrid genome GC content (%)	52.27	52.27	52.27
Total CDS	4,747	4,750	4,750
Coding CDS	4,604	4,604	4,600
tRNA genes	83	86	79
RNA genes	117	120	114
SRA accession no. (Illumina)	SRR24877938	SRR24883437	SRR24883441
SRA accession no. (ONT)	SRR24877936	SRR24883435	SRR24883439
WGS accession no. (GenBank)	JBRYZA000000000	JBRYQL000000000	JBRYZF000000000

The three genomes were assigned to the same pathogen detection cluster, PDS000007781.1237, which includes the MDR persistent strain, REPJJP01, and differentiated the genomes by zero to two SNPs. Hybrid genome assemblies were uploaded to the PubMLST Ribosomal MLST typing database (pubmlst.org), and all were a 100% match to *Salmonella enterica* rST1434 (subspecies *enterica* serovar Newport II). The circular ~47 kb plasmids from all three genomes carried nine AMR genes [*aadA2*, *blaCARB-2*, *dfrA1*, *qnrA1*, *mph(A)*, *floR*, *qacEdelta1*, *sul1*, and *tet(A)*] that matched with antimicrobial susceptibility testing (6). Phylogenetic analyses were previously described (6) and confirmed the close genetic relationship of the three felid *S*. Newport genomes to those from meat, human clinical, environmental, and animal fecal isolates.

## Data Availability

This whole-genome sequencing project has been deposited in NCBI under BioProject number PRJNA860199, with SRA accession numbers SRX16349265, SRX20641647, SRX20641648, SRX20641649, SRX20646762, SRX20646763, SRX20646765, SRX20646766, SRX20646767, and SRX20646768. The post-PGAP sequences are JBRYZA000000000, JBRYQL000000000, and JBRYZF000000000.
